# Numerical Analysis of a Continuous Vulcanization Line to Enhance CH_4_ Reduction in XLPE-Insulated Cables

**DOI:** 10.3390/ma14041018

**Published:** 2021-02-21

**Authors:** Mohd Fuad Anwari Che Ruslan, Dong Joon Youn, Roshan Aarons, Yabin Sun, Shuyu Sun

**Affiliations:** 1 Computational Transport Phenomena Lab, King Abdullah University of Science and Technology, Thuwal 23955-6900, Saudi Arabia; mohdfuadanwari.cheruslan@kaust.edu.sa; 2 Dow Chemical Europe, 8810 Horgen, Switzerland; raarons1@dow.com; 3 Dow Chemical (China) Investment Co., Ltd., Shanghai 201203, China; SYSun@dow.com

**Keywords:** cable insulation, cross-linked polyethylene (XLPE), continuous vulcanization (CV) line, cross-linking reaction, byproduct degassing, reaction selectivity, heat transfer, CH4 diffusion

## Abstract

Herein, we apply a computational diffusion model based on Fick’s law to study the manner in which a cable production line and its operating conditions can be enhanced to effectively reduce the CH${}_{4}$ concentration in cables insulated with cross-linked polyethylene (XLPE). Thus, we quantitatively analyze the effect of the conductor temperature, curing tube temperature distribution, transition zone length, and online relaxation on CH${}_{4}$ generation and transport during the production of 132 kV cables with an insulation thickness of 16.3 mm. Results show that the conductor temperature, which is initially controlled by a preheater, and the curing tube temperature distribution considerably affect the CH${}_{4}$ concentration in the cable because of their direct impact on the insulation temperature. The simulation results show 2.7% less CH${}_{4}$ remaining in the cable when the preheater is set at 160 °C compared with that when no preheater is used. To study the curing tube temperature distribution, we consider three distribution patterns across the curing tube: constant temperature and linear incremental and decremental temperature. The amount of CH${}_{4}$ remaining in the cable when the temperature was linearly increased from 300 to 400 °C was 1.6% and 3.7% lower than in the cases with a constant temperature at 350 °C and a linear temperature decrease from 400 to 300 °C, respectively. In addition, simulations demonstrate that the amount of CH${}_{4}$ removal from the cable can be increased up to 9.7% by applying an elongated and insulated transition zone, which extends the residence time for CH${}_{4}$ removal and decelerates the decrease in cable temperature. Finally, simulations show that the addition of the online relaxation section can reduce the CH${}_{4}$ concentration in the cable because the high cable temperature in this section facilitates CH${}_{4}$ removal up to 2.2%, and this effect becomes greater at low production speeds.

## 1. Introduction

**Figure 1:**
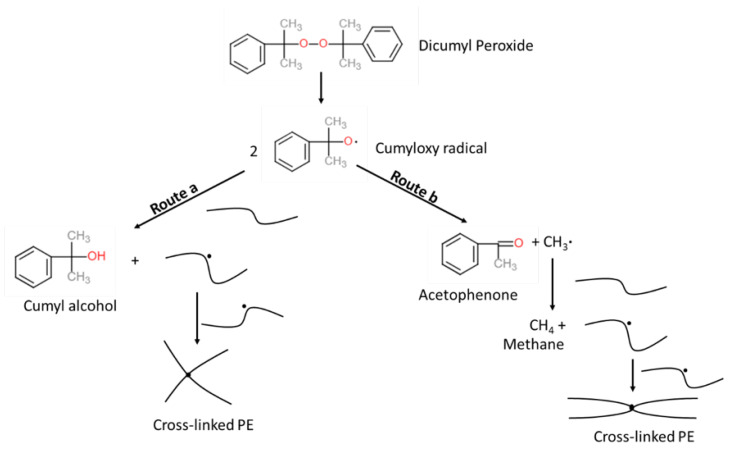
DCP-initiated PE cross-linking reaction scheme [5].

Cross-linked polyethylene (XLPE) is one of the most popular insulation materials for producing power cables due to the exceptional electrical properties, production efficiency, and resistance to chemicals, moisture, and temperature [1,2,3,4,5]. To manufacture and apply XLPE for cable insulation, dicumyl peroxide (DCP) is commonly used as a cross-linking initiator of polyethylene (PE) due to its relatively fast decomposition rate at typical cable manufacturing temperatures, especially in the curing tube [4,6]. However, the production of XLPE also causes obvious issues regarding the creation of its byproduct such as methane (CH${}_{4}$), acetophenone (AP), and cumyl alcohol (CA) (See Figure 1). They not only degrade the performance of the cable, but also raise safety issues mostly because of the flammability of CH${}_{4}$ [1,7,8,9,10,11]. To properly remove such byproducts from the cables, a thermal treatment process, namely byproduct degassing, becomes increasingly important. The byproduct transport phenomenon during the degassing was analyzed experimentally [12,13,14,15,16,17] and numerically [1,4,17,18,19,20,21,22,23]. Among the studies, Youn et al. [4] found that the initial concentration distribution plays a crucial role in determining the degassing efficiency, and the distribution is majorly based on the cable production phase called the continuous vulcanization (CV) process. Based on the previous studies regarding the byproduct transfer and release from the XLPE-insulated cables, Ruslan et al. [5] conducted numerical studies and demonstrated that the cable production conditions in the CV process considerably affect the generation and transport of byproducts within the cables, and therefore, the degassing process should be optimized based on the remaining byproduct after the CV process.

Here, we extend the study to investigate the manner in which the cable production line and operating conditions can be enhanced to effectively reduce the concentration of byproducts in an XLPE-insulated cable during production, and therefore to minimize the cost for the byproduct degassing. From the previous CV line study [5], we figured that the temperature is the key factor for both the creation and transport of the byproducts so that the key elements in the CV line process affecting the temperature of the cable were chosen and customized in this paper such as the conductor pre-heater, curing tube temperature control, transition zone length and insulation, and online relaxation between the cooling sections. The details of the elements are described in Section 2. On top of the governing equations and boundary conditions applied for the basic CV line process in our previous work [5], additional conditions have to be implemented for the elements tested in this work, and the numerical models and boundary conditions are demonstrated in Section 3. In Section 4, we show how the case studies are designed based on the basic CV line configuration to possibly enhance the cable production process to minimize the byproduct remaining in the cable. The essential simulation data such as temperature distribution and byproduct concentration in the cable and conclusion are summarized in Section 5 and Section 6, respectively.

## 2. Cable Production Conditions

**Figure 2:**
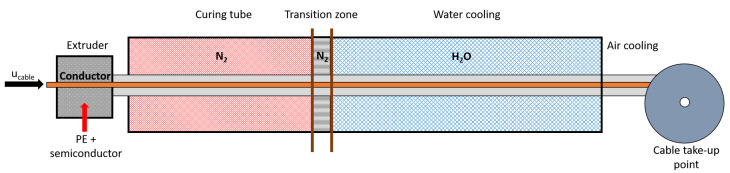
Schematic of typical power cable production line.

Figure 2 shows the schematic of the general cable production line. The cable production line, namely a continuous vulcanization (CV) line, comprises the extrusion, heating, and cooling processes. In the extruder, the inner semiconductor (SC), PE insulation, and outer SC layers are applied simultaneously over the preheated conductor, and the process is called triple extrusion. The process is to create smooth interfaces between the cable layers, which is essential to prevent concentrated electrical stress and reduce contamination during extrusion [24,25]. The cable then moves into the curing tube, where the cable is heated primarily via radiation from the tube wall. Most of the cross-linking reaction occurs in this section because the high temperature promotes the peroxide initiator decomposition. The tube, which is filled with high-pressure nitrogen, includes several heating zones with the temperatures of each heating zone able to be regulated for optimal production. There is a short section filled with high-pressure, unheated nitrogen between the curing tube and the cooling stages. This section is commonly referred to as the transition zone. In this stage, radiation heat transfer occurs between the high-temperature cable and the transition zone casing, which is typically exposed to ambient air. After exiting the transition zone, the cable is cooled in two cooling stages. In the first stage, the cable is cooled through forced convection heat transfer with water, typically flowing in the opposite direction of the cable production. In the second stage, the cable is cooled via natural convection heat transfer through ambient air until the cable reaches the take-up point and is securely bound on a reel.

**Figure 3:**
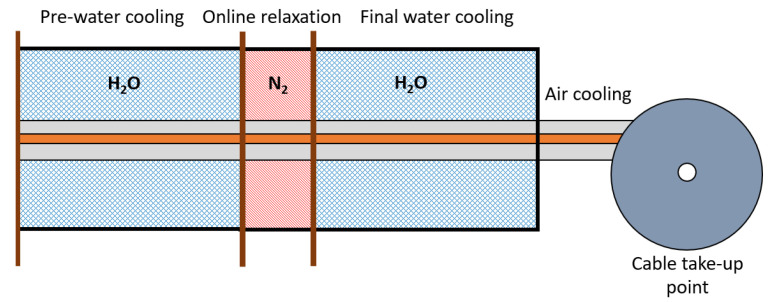
Schematic of the cooling section for the power cable production line with online relaxation.

In this study, we also considered the effect of the online relaxation section on byproduct transport. The online relaxation section is a heating section optionally added in the middle of the cooling section (see Figure 3) to minimize the mechanical stress unwillingly caused by the rapid temperature drop in the insulation layer [26]. With the online relaxation process, the cable insulation temperature temporarily increases again, which might offer another window to release CH${}_{4}$ from the cable. Thus, a quantitative comparison in terms of the remaining CH${}_{4}$ was conducted in this part of the study with and without an online relaxation in the CV line processes. The design of this section and its heat transfer mechanism are similar to those associated with the heating zones of the curing tube.

## 3. Mathematical Model

The generation and transport of the cross-linking byproducts are calculated by the computational model, and the overall procedure was described in our previous paper [5]. In this section, therefore, we discuss only the main assumptions and equations used to simulate the cross-linking reaction, heat transfer, and the byproduct transport process on top of the additional equations and boundary conditions for the additional features (e.g., conductor pre-heater and online relaxation) implemented in this study.

The main assumptions on which the model is based are [5]: (1) the cable layers are homogeneous and isotropic in terms of thermal conductivity and byproduct diffusion; (2) DCP is uniformly distributed along the entire insulation; (3) the heat generated by the cross-linking reaction, i.e., 900 kJ·kg${}^{-1}$ of DCP, is negligible when compared with the heat supplied by the heating zones [27,28]; this cross-linking reaction enthalpy is, therefore, not included in the model; (4) because the cable has no free space between the layers, there is no extra loss of thermal energy and the byproduct concentration along the interfaces.

### 3.1. Cross-Linking Reaction

Typically, a cross-linking reaction is defined in terms of peroxide conversion α instead of peroxide concentration. Equation (1) shows the relations between α and peroxide concentration.

(1) $$\alpha =1-\frac{c_{dcp}}{c_{dcp}^{inlet}}$$

where $c_{dcp}$ is the DCP concentration and the superscript inlet indicates the location of the curing tube inlet. For steady-state production, the DCP transport, defined in terms of α, can be computed as follows:

(2) $$-u\cdot \nabla \left(\alpha c_{dcp}^{inlet}\right)=r_{p}$$

where *u* is the cable production speed and $r_{p}$ is the DCP decomposition rate. In this study, $r_{p}$ is modeled in terms of first-order kinetics, as shown in Equation (3).

(3) $$r_{p}=-A_{p}\exp\left(\frac{-E_{a,p}}{RT}\right)\left(1-\alpha \right)c_{dcp}^{inlet}$$

where $A_{p}$ and $E_{a,p}$ are the pre-exponential factor and activation energy for the DCP decomposition reaction, respectively.

As shown in Figure 1, the DCP decomposition reaction produces the cumyloxy radical, which then can either proceed with hydrogen abstraction reaction (route *a*) or β-scission reaction (route *b*). The hydrogen abstraction reaction (route *a*) produces a byproduct, CA, while the β-scission reaction (route *b*) produces two byproducts, AP and CH${}_{4}$. The reaction selectivity of route *a* and route *b* reactions is used to estimate the amount of the byproducts generated from the reactions. The reaction selectivity $S_{b}$ of route *b* is defined as in Equation (4).

(4) $$S_{b}=\frac{r_{b}}{r_{a}+r_{b}}$$

where $r_{a}$ and $r_{b}$ are the reaction rates for routes *a* and *b*, respectively, and are defined as follows:

(5) $$r_{a}=A_{a}\exp\left(\frac{-E_{a,a}}{RT}\right)c_{ph}\left(2\alpha c_{dcp}^{inlet}\right)$$

(6) $$r_{b}=A_{b}\exp\left(\frac{-E_{a,b}}{RT}\right)\left(2\alpha c_{dcp}^{inlet}\right)$$

where $A_{a}$ and $A_{b}$ are the respective pre-exponential factors for the reactions of routes *a* and *b*, $E_{a,a}$ and $E_{a,b}$ are the respective activation energies for the reactions of routes *a* and *b*, and $c_{ph}$ is the ethylene monomer concentration. Based on the reaction rate defined in Equations (5) and (6), Equation (4) can be expanded as in Equation (7).

(7) $$S_{b}=\frac{A_{b}\exp\left(\frac{-E_{a,b}}{RT}\right)}{A_{a}\exp\left(\frac{-E_{a,a}}{RT}\right)c_{ph}+A_{b}\exp\left(\frac{-E_{a,b}}{RT}\right)}$$

### 3.2. Heat Transfer

**Figure 4:**
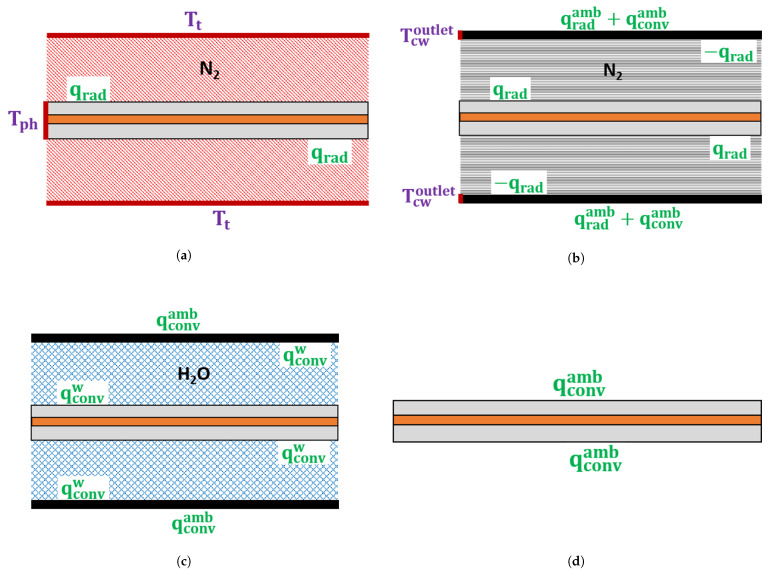
Schematic of the heat transfer model in the (**a**) heating zone, (**b**) transition zone, (**c**) water-cooling section, and (**d**) air-cooling section.

The temperature profile for each component (conductor, insulation, nitrogen, water, casing wall) in a steady-state production process can be calculated using Equation (8).

(8) $$\rho C_{p}u\cdot \nabla T=\nabla \cdot \left(k\nabla T\right)$$

where ρ is the density, $C_{p}$ is the specific heat capacity, and *k* is the thermal conductivity. For each CV line section, Figure 4 presents the heat exchange between the cable and remaining components surrounding the cable. The following subsections explain the boundary conditions and equations used to model the heat transfer for each CV line section.

#### 3.2.1. Heating Section

The radiation heat flux $q_{rad}$ along the cable surface can be computed as follows:

(9) $$q_{rad}=\frac{\sigma (T_{t}^{4}-T_{c}^{4})}{\frac{1}{\epsilon_{c}}+\left(\frac{d_{c}}{d_{t}}\right)\left(\frac{1}{\epsilon_{t}}-1\right)}$$

where σ is the Stefan–Boltzmann constant, $T_{t}$ is the wall temperature of the heating section, $T_{c}$ is the cable temperature, $d_{c}$ is the cable diameter, $d_{t}$ is the heating section diameter, $\epsilon_{c}$ is the cable surface emissivity, and $\epsilon_{t}$ is the wall emissivity of the heating section. We consider $\epsilon_{c}=0.7$ and $\epsilon_{t}=1.0$, as per Shugai and Yakubenko [28]. In Equation (9), $T_{t}=T_{hw}$, which is the wall temperature of the heating section.

The boundary condition applied at the curing tube inlet is shown in Equation (10).

(10) $$T_{c}^{inlet}=T_{ph}$$

where $T_{ph}$ is the preheated temperature of the cable components, which is separately controlled for each component, and the superscript inlet indicates the curing tube inlet location.

#### 3.2.2. Transition Zone

The casing wall can be a crucial part of the cable temperature calculation because the wall blocks the direct contact of the cable with ambient air and could be an additional source or sink of the heating energy in the cable. Therefore, we compute the temperature distribution of the casing wall in the transition zone through conduction while considering the heat dissipation to ambient air. The boundary condition applied at the transition zone inlet is shown in Equation (11).

(11) $$T_{tw}^{inlet}=T_{cw}^{outlet}$$

where $T_{tw}^{inlet}$ is the wall temperature at the transition zone inlet and $T_{cw}^{outlet}$ is the wall temperature at the outlet of the curing tube. Because $T_{tw}^{inlet}$ is considerably greater than the ambient temperature $T_{amb}$, a significant amount of heat is dissipated through radiation and natural convection to ambient air. The radiation heat flux to ambient air, $q_{rad}^{amb}$, can be obtained using Equation (12) [29], whereas the convective heat flux to ambient air, $q_{conv}^{amb}$, can be obtained using Equation (13).

(12) $$q_{rad}^{amb}=\sigma \epsilon_{t}\left(T_{tw}^{4}-T_{amb}^{4}\right)$$

(13) $$q_{conv}^{amb}=h\left(T_{tw}-T_{amb}\right)$$

In Equation (13), *h* is the convective heat transfer coefficient.

In addition, the radiation heat flux from the casing wall to the cable can be calculated using Equation (9) with $T_{tw}=T_{t}$.

#### 3.2.3. Cooling Section

In the water-cooling section, the cooling water directly contacts the cable surface while flowing in the opposite direction. Hence, the major heat transfer mechanism in this section is forced convection. The convective heat flux from the cable surface to water, $q_{conv}^{w}$, can be calculated as follows:

(14) $$q_{conv}^{w}=h\left(T_{c}-T_{w}^{ave}\right)$$

where $T_{w}^{ave}$ is the average water temperature.

Furthermore, the convective heat flux between the casing and cooling water can be an important source of the overall temperature changes, and thus, the heat transfer is included and calculated in the simulation using Equation (14). The heat dissipation through natural convection from the casing wall to ambient air is computed using Equation (13).

The heat transfer via natural convection between the cable and ambient air in the air-cooling section can be calculated using Equation (13).

### 3.3. Byproduct Transport

For a steady-state cable production, CH${}_{4}$ transport can be computed as follows:

(15) $$\nabla \cdot \left(-D\nabla c_{m}\right)+u\cdot \nabla c_{m}=r_{m}$$

where *D* is the diffusion coefficient, $c_{m}$ is the CH${}_{4}$ concentration, and $r_{m}$ is the rate of CH${}_{4}$ generation. As in Equation (16), $r_{m}$ is dependent on the selectivity, $S_{b}$ of the reaction route *b*, which is defined in Equation (7), and the cross-linking reaction rate, $r_{p}$, which is defined in Equation (3).

(16) $$r_{m}=2S_{b}r_{p}c_{dcp}^{inlet}$$

The Arrhenius expression shown in Equation (17) is used to define the diffusion coefficient, *D* [17,22].

(17) $$D=A_{d}\exp\left(-\frac{E_{a,d}}{RT}\right)$$

where $A_{d}$ and $E_{a,d}$ are the pre-exponential factor and activation energy, respectively. Zero CH${}_{4}$ concentration is considered to be the boundary condition at the CV line inlet and along the cable surface.

## 4. Simulation Parameters

### 4.1. Cable and CV Line Specifications

**Figure 5:**
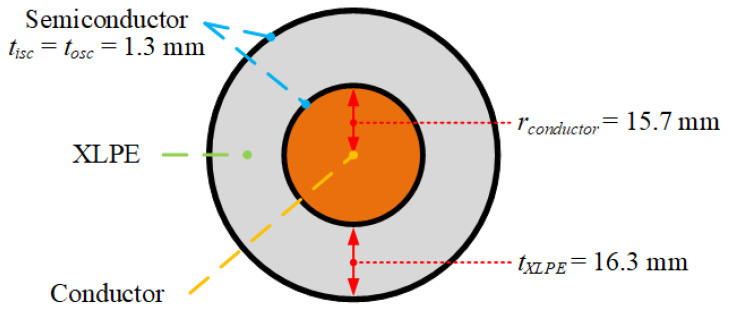
132 kV cable specification [5].

We analyze a representative byproduct transport scenario for a single-core 132 kV cable with a typical XLPE insulation configuration (16.3 mm thick) based on our previous work [5]. Figure 5 presents the schematic of this cable. The diameter and casing wall thickness throughout the CV line were set to 200 mm and 10 mm, respectively. Additionally, Table 1 shows the dimensions taken from Kosar and Gomzi [30] for each of the CV line sections.

Table 2 presents the CV line operating conditions applied in this study. The ambient temperature, $T_{amb}$, is 25 °C.

### 4.2. Cross-Linking Reaction

Table 3 presents the Arrhenius parameters used for modeling the cross-linking, hydrogen abstraction, and β-scission reactions in this study. We used the reaction parameters computed for reactions in chlorobenzene and cumene [31] as estimates for the β-scission reaction in PE. This estimation is in accordance with the study by Avila et al. [32], which showed that the β-scission reaction involving non-polar solvents is primarily a function of temperature.

**Figure 6:**
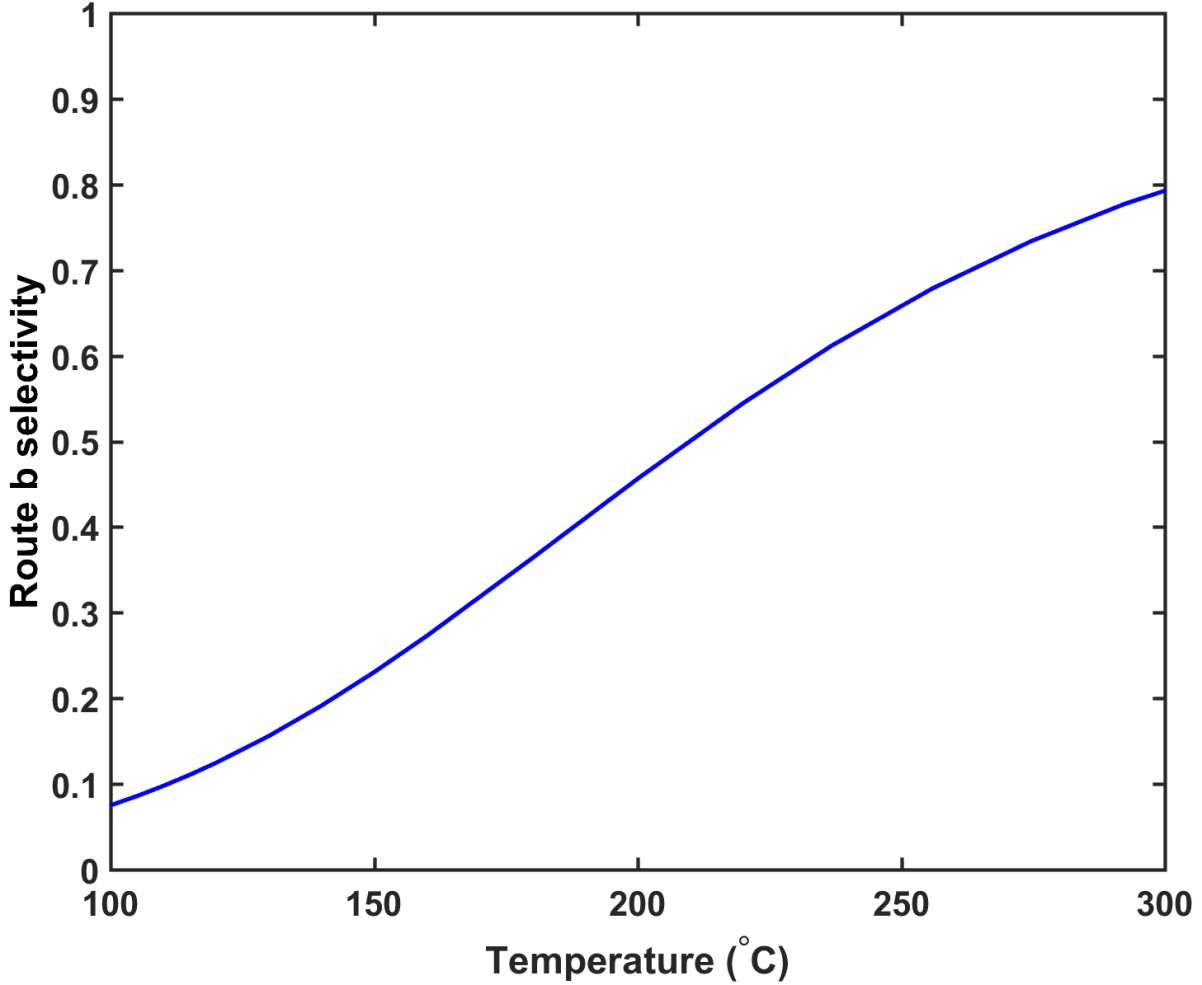
Route *b* reaction selectivity profile estimated by the kinetic parameters in Rado et al. [33].

The kinetic parameters for the hydrogen abstraction reaction were estimated based on the kinetic parameters for the β-scission reaction and on the byproduct ratio data for DCP decomposition in PE [33], and Equation (18) is used for this estimation.

(18) $$\ln\frac{c_{ca}}{c_{ap}c_{ph}}=\ln\frac{A_{a}}{A_{b}}-\frac{E_{a,a}-E_{a,b}}{RT}$$

where $c_{ca}$ and $c_{ap}$ are the CA and AP concentrations, respectively. Figure 6 demonstrates the selectivity profile of the route *b* reaction, representing that the selectivity of route *b* positively correlates with the curing temperature. This trend is consistent with the DCP decomposition data in other solvents [35,36,37]. In this study, the DCP concentration was set to 2 wt%.

### 4.3. Thermophysical Properties

Table 4 presents the thermal and mechanical properties considered for each cable component in this study.

### 4.4. CH${}_{4}$ Diffusion

**Figure 7:**
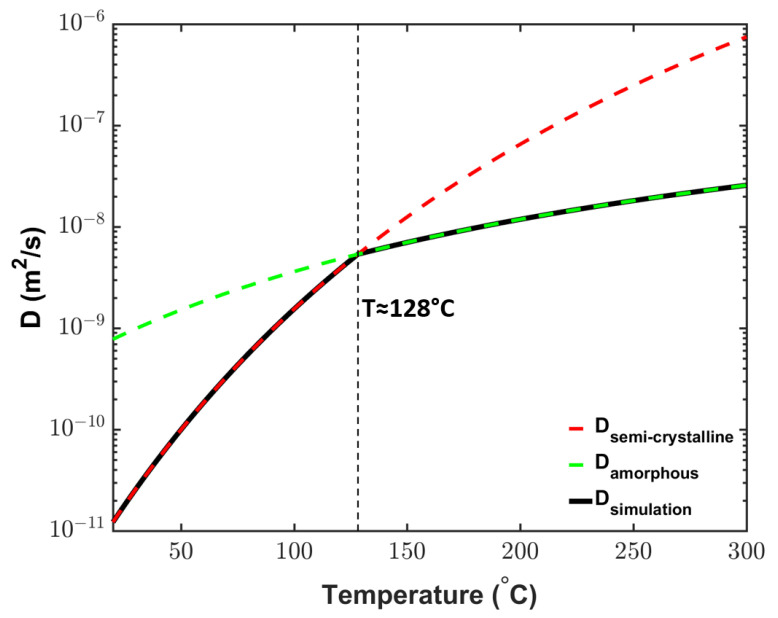
CH${}_{4}$ diffusion coefficient variations in XLPE as semi-crystalline (red line) and amorphous (green line) phases of the XLPE, which were then combined to represent the diffusion coefficient variation applied in the simulation (black line) [5]. The kinetic parameters for the red and green lines are from Sun and Person [22] and Yang et al. [41], respectively.

The cable temperature changes considerably during production. In the curing tube, the cable temperature is considerably above the PE melting point, whereas the cable typically is at ambient temperature at the cable take-up point. This change in temperature invokes a phase transition in XLPE during cable production. At the ambient temperature, XLPE has a semicrystalline structure, but the crystallites begin to melt at approximately 70 °C [1]. Because the semicrystalline structure may considerably affect the diffusion within XLPE [38,39,40], the effect of phase changes on the diffusion coefficient must be considered to ensure accurate byproduct transport analysis. As shown in Figure 7, thus, we use the experimentally measured diffusion coefficient profile for semicrystalline XLPE at low temperatures [22] and that for the amorphous phase estimated via molecular dynamic simulations [41] at high temperatures. The two diffusion profiles intersect at approximately 128 °C, indicating the transition temperature between the two XLPE phases in this study.

In addition to temperature and crystallinity, CH${}_{4}$ diffusion could be influenced by other parameters, including the cross-linking density and CH${}_{4}$ concentration. However, a previous molecular dynamic simulation study [41] showed that only a small amount of cross-linking density is observed under cable production conditions (about 4.2% for production with 2.5% peroxide content), and under these conditions, cross-linking density has a negligible effect on CH${}_{4}$ diffusion. Furthermore, Dutta and Bhatia used molecular dynamic simulation to show that CH${}_{4}$ concentration have a negligible effect on CH${}_{4}$ diffusion [42].

An inverse calculation conducted by Youn et al. [23] using a set of experimental CH${}_{4}$ concentration data indicated that the conductor also played an essential role in the byproduct transfer because of its relatively large diffusion coefficient. Therefore, the cable conductor is included in the CH${}_{4}$ diffusion simulations. The diffusion coefficient of the cable conductor is obtained based on the results of Youn et al. [23] and is approximately 41.56% of that of XLPE.

## 5. Results and Discussion

### 5.1. Overview of the Case Studies

We conducted numerical simulations to determine the manner in which the cable production line and operating conditions can be enhanced to effectively reduce the CH${}_{4}$ concentration during cable production. The condition for >99.9% cross-linking was applied to determine the production speed in all case studies.

### 5.2. Conductor Temperature Study

**Figure 8:**
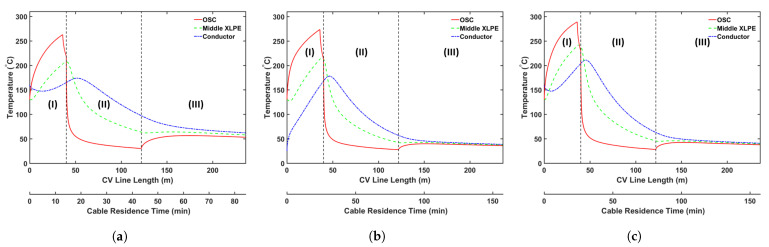
Temperature profile across the CV line for the conductor temperature study: (**a**) Case 1a, (**b**) Case 1b, and (**c**) Case 1c. Segments (I)–(III) correspond to the curing tube and transition zone, the water-cooling segment, and the air-cooling segment, respectively.

**Figure 9:**
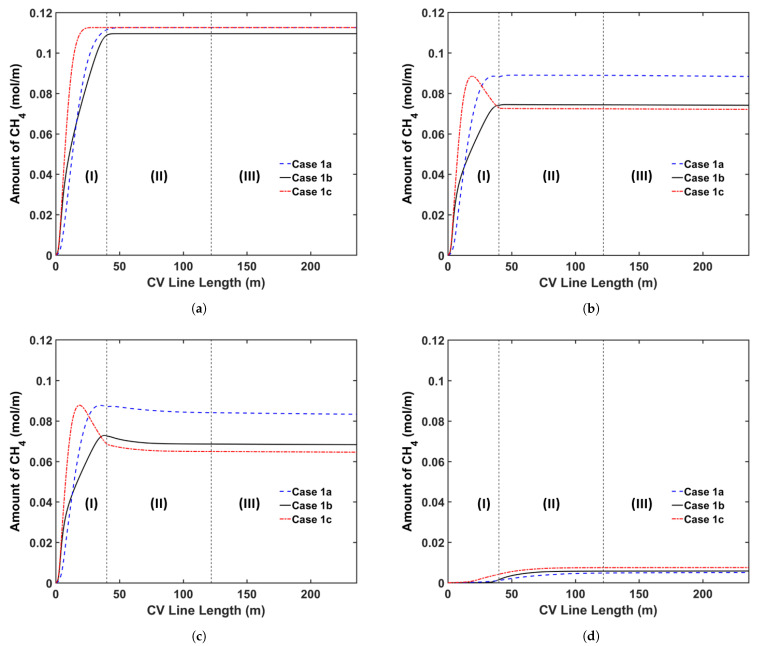
Axial CH${}_{4}$ concentration profile across the CV line for the conductor temperature study: (**a**) total CH${}_{4}$ generated, (**b**) CH${}_{4}$ concentration in the entire cable, (**c**) CH${}_{4}$ concentration in XLPE+SC, and (**d**) CH${}_{4}$ concentration in the conductor. Segments (I)–(III) correspond to the curing tube and transition zone, the water-cooling segment, and the air-cooling segment, respectively.

**Figure 10:**
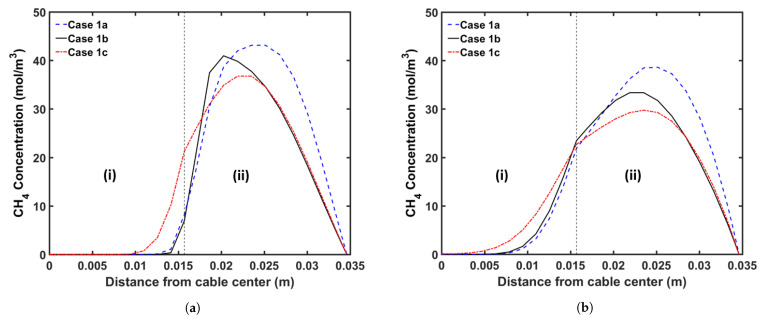
Radial CH${}_{4}$ concentration profile for the conductor temperature study at (**a**) the curing tube exit and (**b**) the cable take-up point. Segment (i) corresponds to the conductor, and Segment (ii) corresponds to the XLPE and SC layers.

We begin by analyzing the manner in which the cable conductor temperature affects CH${}_{4}$ transport. Typically, the cable conductor is heated by the preheater to the temperature up to 160 °C before extrusion. This heating process reduces the heat transferred from the insulation layer to the cable conductor during heating in the curing tube. Because the cross-linking reaction rate increases exponentially with the temperature, we consider a high conductor temperature, thereby ensuring that the cable can be produced at a high production speed without changing the heating conditions in the curing tube. It is important to understand the effect of varying the conductor temperature on CH${}_{4}$ generation and transport because the cross-linking reaction rate, reaction selectivity, and CH${}_{4}$ diffusion are considerably dependent on temperature. Table 5 presents the case studies considered in this analysis. In the case of conductor temperatures, we considered the ambient temperature (25 °C) and a temperature of 160 °C. In Cases 1a and 1b, the maximum production speed that yields >99.9% cross-linking was considered. In addition, Case 1c was designed to determine the effect of different conductor temperatures at similar production speeds. In each case, we maintained the curing tube temperature at 350 °C and set the insulation temperature at the curing tube inlet at 130 °C. Figure 8 shows the temperature profiles, whereas Figure 9 and Figure 10 show the axial and radial CH${}_{4}$ concentration profiles, respectively. Table 6 indicates the amount of CH${}_{4}$ present in the cable at the cable take-up point, and %CH${}_{4}$ stands for the fractional comparison between the cases regarding the remaining CH${}_{4}$ in the entire cable.

Results show that the amount of CH${}_{4}$ present in Case 1a was 19.2% higher than that in Case 1b at the cable take-up point. This significant difference can be attributed to the combined effect of high insulation temperature and high production speed. In Case 1a, the insulator temperature increases more rapidly than that in Case 1b, resulting in the increased generation of CH${}_{4}$ in Case 1a. In Case 1a, 2.7% more CH${}_{4}$ is generated than that in Case 1b. This result is consistent with the selectivity trend of the cross-linking reaction shown in Figure 6. The high production speed of Case 1a (86.7% faster than Case 1b) contributes to this significant difference. The short residence time in Case 1a considerably limits the CH${}_{4}$ removal from the cable; therefore, 21.5% of the total CH${}_{4}$ generated is removed from the cable in Case 1a versus 32.3% in Case 1b.

At similar production speeds, a higher conductor temperature results in less CH${}_{4}$ in the cable at the cable take-up point; 2.7% less CH${}_{4}$ is present in Case 1c when compared with that in Case 1b. Although the high temperature increases CH${}_{4}$ generation in the insulation layer and CH${}_{4}$ removal from the cable, in this case, its effect on CH${}_{4}$ removal is more significant than that on CH${}_{4}$ generation. In Case 1b, 2.8% less CH${}_{4}$ is generated than that in Case 1c. Further, 32.3% of the total CH${}_{4}$ generated is removed in Case 1b versus 36.0% in Case 1c. In addition, the high conductor temperature in Case 1c facilitates the diffusion of CH${}_{4}$ into the conductor, increasing the amount of CH${}_{4}$ in the conductor in Case 1c compared with that in Case 1b.

### 5.3. Curing Tube Temperature Distribution Study

Another parameter considered in this study involves the heating conditions associated with the curing tube. As mentioned in Section 2, the curing tube includes several heating zones, and the temperature of each heating zone can be adjusted individually for optimal production. The effect of the application of different curing tube temperature distributions on CH${}_{4}$ generation and transport must be understood because the cross-linking reaction rate and selectivity and CH${}_{4}$ diffusion are considerably dependent on temperature.

**Figure 11:**
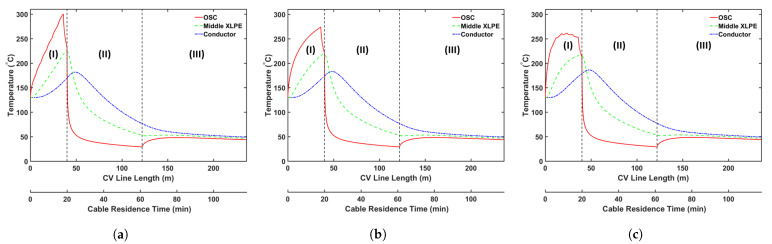
Temperature profile across the CV line for the curing tube temperature distribution study: (**a**) Case 2a, (**b**) Case 2b, and (**c**) Case 2c. Segments (I)–(III) correspond to the curing tube and transition zone, the water-cooling segment, and the air-cooling segment, respectively.

**Figure 12:**
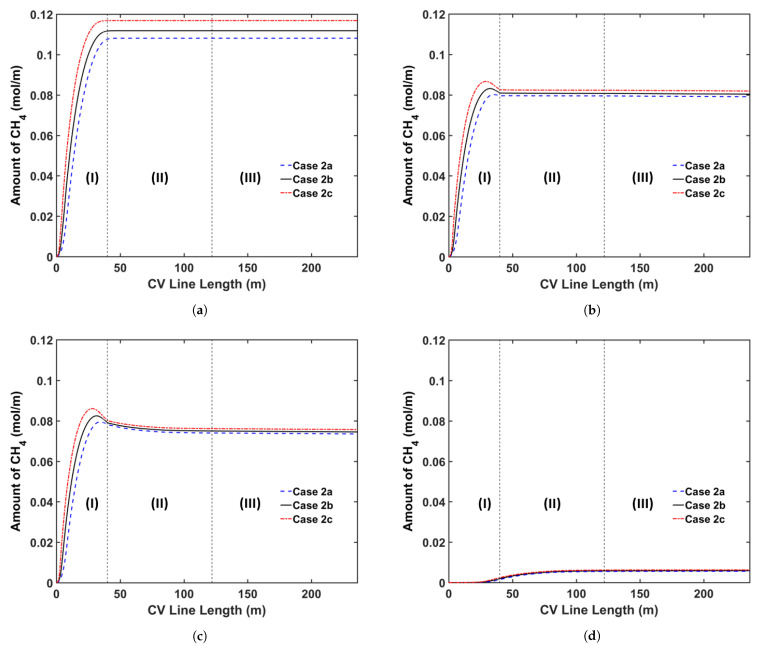
Axial CH${}_{4}$ concentration profile across the CV line for the curing tube temperature distribution study: (**a**) total CH${}_{4}$ generated, (**b**) CH${}_{4}$ concentration in the entire cable, (**c**) CH${}_{4}$ concentration in XLPE+SC, and (**d**) CH${}_{4}$ concentration in the conductor. Segments (I)–(III) correspond to the curing tube and transition zone, the water-cooling segment, and the air-cooling segment, respectively.

**Figure 13:**
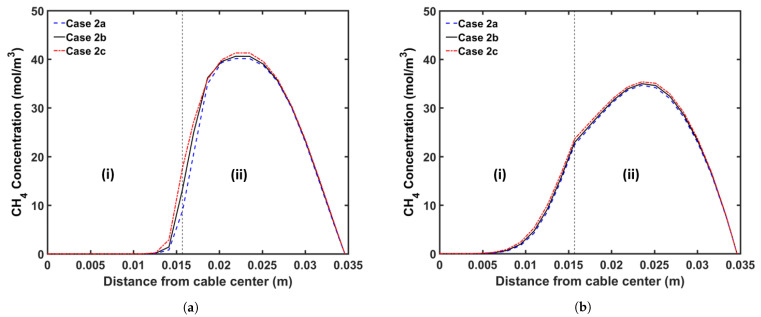
Radial CH${}_{4}$ concentration profile for the curing tube temperature distribution study at (**a**) the curing tube exit and (**b**) the cable take-up point. Segment (i) corresponds to the conductor, and Segment (ii) corresponds to the XLPE and SC layers.

We consider three curing tube temperature distribution strategies in this analysis: (a) constant temperature, (b) linear temperature increase, and (c) linear temperature decrease. In this study, we consider a curing tube with six heating zones. Table 7 presents the case studies considered in this analysis. The average curing tube temperature for each case is kept at 350 °C. The production speed is 2.0 m/min, and the operating conditions of each case study are listed in Table 2. Figure 11 shows the temperature profiles, whereas Figure 12 and Figure 13 show the axial and radial CH${}_{4}$ concentration profiles, respectively. Table 8 indicates the remaining amount of CH${}_{4}$ in the cable at the cable take-up point.

Results show that the curing tube temperature distribution considerably affects CH${}_{4}$ generation and transport. The insulation temperature in Case 2c increases more rapidly than in Cases 2a and 2b, and the resulting high insulation temperature results in the generation of the highest amount of CH${}_{4}$ in Case 2c, consistent with the reaction selectivity trend shown in Figure 6. Case 2c generates 4.4% and 7.5% more CH${}_{4}$ than Cases 2b and 2a, respectively. The rapid temperature increase in the insulation also indicates that the largest fraction of CH${}_{4}$ is removed from the cable in Case 2c. Approximately 29.9% of the total CH${}_{4}$ generated is removed from the cable in Case 2c when compared with 28.1% and 26.8% in Cases 2b and 2a, respectively. In addition, although Cases 2a and 2b have a higher maximum cable temperature than Case 2c, the effect on CH${}_{4}$ removal from the cable is not significant because the maximum temperature was achieved near the end of the curing tube section. Results show that Case 2a, which generates the least amount of CH${}_{4}$, but also removes the least amount of CH${}_{4}$ from the cable, has the lowest amount of CH${}_{4}$ in the cable at the cable take-up point. In Case 2a, 1.6% and 3.7% less CH${}_{4}$ can be observed in the cable at the cable take-up point when compared to Cases 2b and 2c, respectively.

### 5.4. Transition Zone Study

**Figure 14:**
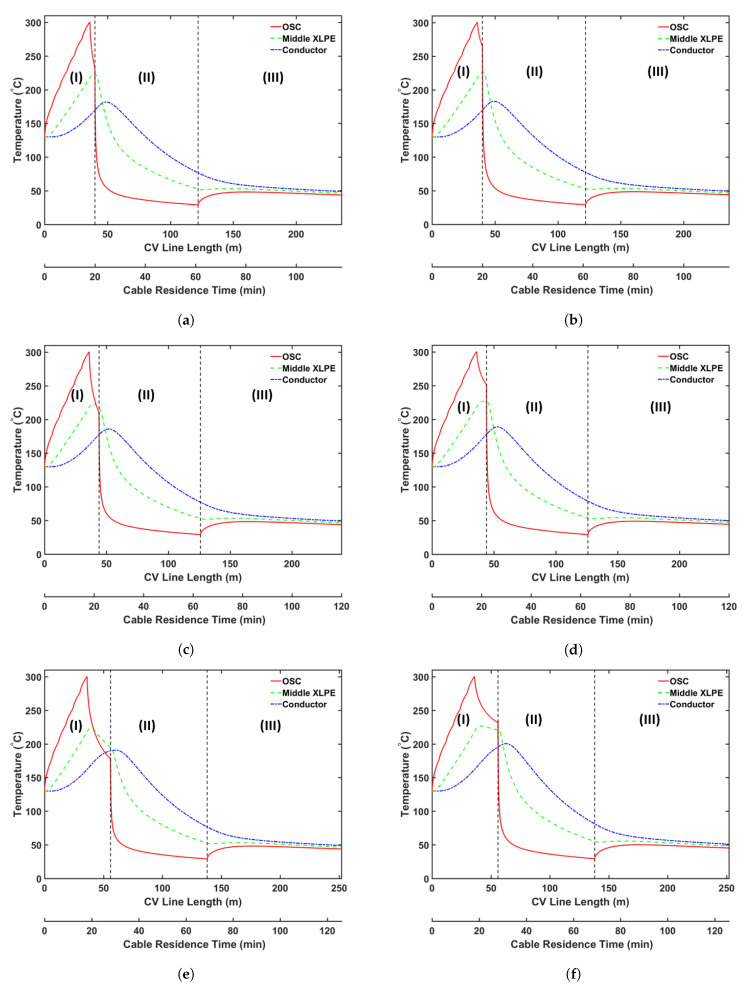
Temperature profile across the CV line for the transition zone study: (**a**) Case 3a, (**b**) Case 3b, (**c**) Case 3c, (**d**) Case 3d, (**e**) Case 3e, and (**f**) Case 3f. Segments (I)–(III) correspond to the curing tube and transition zone, the water-cooling segment, and the air-cooling segment, respectively.

**Figure 15:**
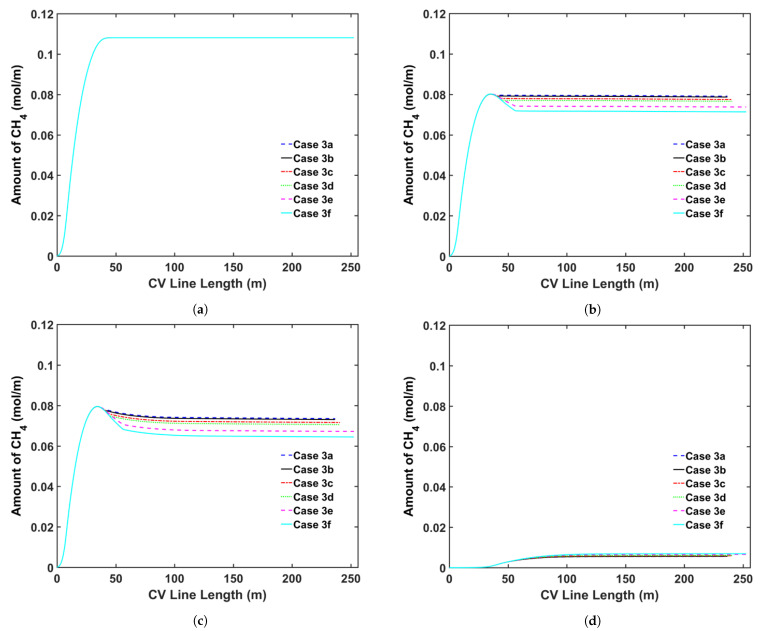
Axial CH${}_{4}$ concentration profile across the CV line for the transition zone study: (**a**) total CH${}_{4}$ generated, (**b**) CH${}_{4}$ concentration in the entire cable, (**c**) CH${}_{4}$ concentration in XLPE + SC, and (**d**) CH${}_{4}$ concentration in the conductor.

**Figure 16:**
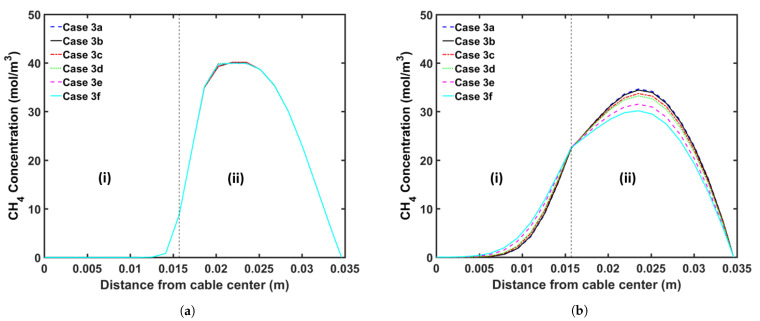
Radial CH${}_{4}$ concentration profile for the transition zone study at (**a**) the curing tube exit and (**b**) the cable take-up point. Segment (i) corresponds to the conductor, and Segment (ii) corresponds to the XLPE and SC layers.

In our previous study [5], we showed that CH${}_{4}$ was removed from the cable mainly in the curing tube and the transition zone because CH${}_{4}$ removal from the cable is limited in the cooling section owing to the considerably low temperature of the cable surface. Thus, modifying the parameters of the transition zone is an alternative to increase CH${}_{4}$ removal during the cable production. Herein, we analyzed the effect of the following transition zone parameters on CH${}_{4}$ removal from the cable: (a) transition zone length and (b) thermal insulation at the transition zone wall. Table 9 presents the case studies used in this analysis. We consider cable production with a transition zone up to 20 m long to study how the length of the transition zone affects CH${}_{4}$ transport. We apply the heating conditions of Case 2a and the operating conditions listed in Table 2 for each case study. Figure 14 shows the temperature as a function of the length of the CV line, whereas Figure 15 and Figure 16 show the axial and radial CH${}_{4}$ concentrations, respectively. Table 10 presents the remaining concentration of CH${}_{4}$ in the cable at the cable take-up point.

Simulation results show that CH${}_{4}$ removal from the cable can be enhanced by increasing the length of the transition zone and applying thermal insulation to the wall of the transition zone. This phenomenon is mostly due to the fact that a significant amount of CH${}_{4}$ is removed while the cable goes through the transition zone, and the residence time in the section is extended when the length of the transition zone is increased. The thermal insulation applied on the transition zone slows the decrease in the cable temperature in the transition zone. Therefore, the additional thermal insulation facilitates CH${}_{4}$ removal from the cable because CH${}_{4}$ diffusion increases exponentially with temperature. Furthermore, the effect of thermal insulation on CH${}_{4}$ removal becomes more apparent with the increasing length of the transition zone. Overall, CH${}_{4}$ removal from the cable can be increased by up to 9.7% by increasing the length of the transition zone and applying thermal insulation to the wall of the transition zone.

### 5.5. Online Relaxation Study

**Figure 17:**
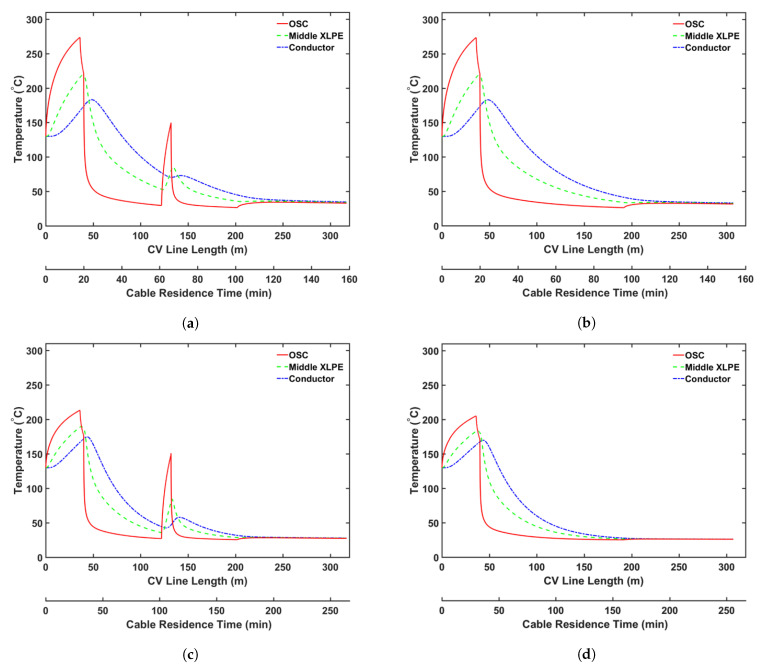
Temperature profile across the CV line for the online relaxation study: (**a**) Case 4a, (**b**) Case 4b, (**c**) Case 4c, and (**d**) Case 4d.

**Figure 18:**
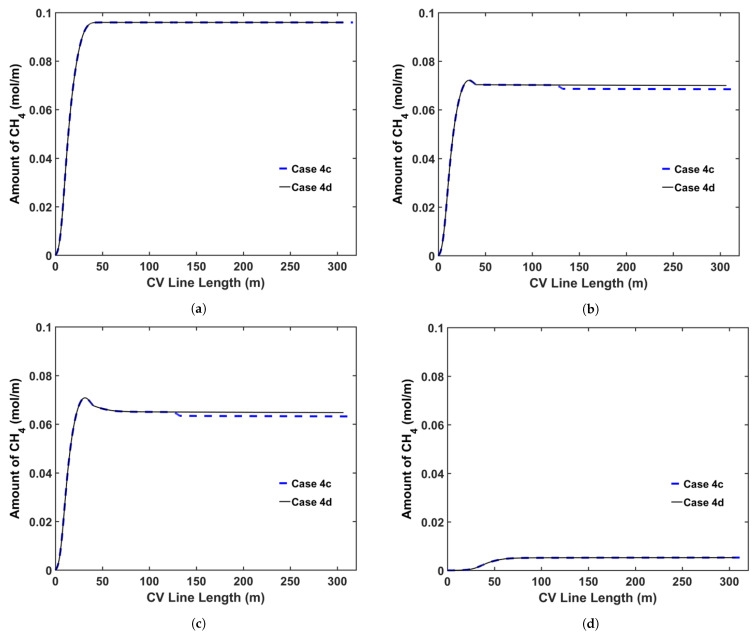
Axial CH${}_{4}$ concentration profile across the CV line for the online relaxation study (Case 4c and Case 4d): (**a**) total CH${}_{4}$ generated, (**b**) CH${}_{4}$ concentration in the entire cable, (**c**) CH${}_{4}$ concentration in XLPE+SC, (**d**) CH${}_{4}$ concentration in the conductor.

**Figure 19:**
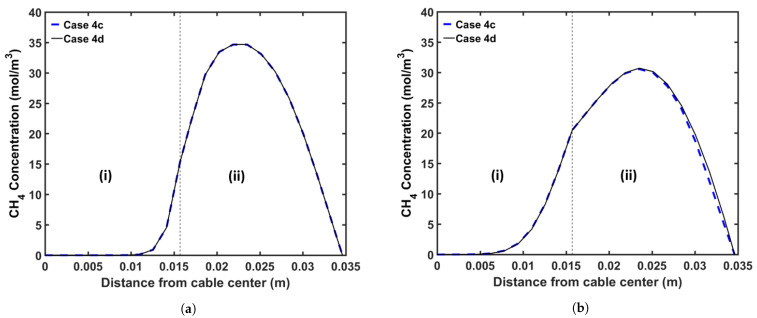
Radial CH${}_{4}$ concentration profile for the online relaxation study (Case 4c and Case 4d) at (**a**) the curing tube exit and (**b**) the cable take-up point. Segment (i) corresponds to the conductor, and Segment (ii) corresponds to the XLPE and SC layers.

We also considered the effect of additional heating in the online relaxation section on CH${}_{4}$ removal from the cable during production. Table 11 presents the parameters for the CV line associated with this analysis. We consider two operating conditions, representing high- and low-speed production. Table 12 presents the case studies used in this analysis. A constant temperature is imposed across the section for cases including the online relaxation section. The temperature of this section is fixed based on the condition that the cable surface exiting from this section exhibits a temperature of approximately 150 °C. We apply the operating conditions listed in Table 2 for each case study. Figure 17 shows the temperature profiles for all cases, and Figure 18 and Figure 19 show the axial and radial CH${}_{4}$ concentration profiles for Cases 4c and 4d, respectively. Finally, Table 13 presents the remaining concentration of CH${}_{4}$ in the cable at the cable take-up point.

As presented in Table 13, the high temperature at the cable surface in the online relaxation section shows a positive effect on CH${}_{4}$ removal from the cable. However, the effect of the online relaxation on the CH${}_{4}$ removal was relatively minor compared with the other CV line production conditions shown in the previous case studies. As shown in Figure 17, the online relaxation majorly affects only the temperature of the external side of the cable due to the relatively short residence time within the section. In the later process of the CV line such as the cooling parts, however, a relatively large amount of CH${}_{4}$ has already been transferred from the XLPE insulation into the conductor, and therefore, the amount of CH${}_{4}$ that can be influenced by the temperature increase due to the online relaxation is also limited accordingly. Therefore, the effect of this online relaxation heating is more apparent at low production speeds because the cable residence time in the online relaxation section is longer than that at high production speeds, and thus, a temperature increase effect can be extended further toward the center of the cable. For Cases 4a and 4c, specifically, 1.4% and 2.2% less CH${}_{4}$ is shown in the cable when compared with those in Cases 4b and 4d, respectively.

## 6. Conclusions

In this study, we used a computational diffusion model to study the manner in which the CV line and operating conditions can be enhanced to effectively reduce the CH${}_{4}$ concentration in an XLPE-insulated cable during production. We analyzed the effect of the conductor temperature, curing tube temperature distribution, transition zone length, and online relaxation section on CH${}_{4}$ generation and transport when producing 132 kV cables with 16.3 mm thick insulation. Results indicate that the cable conductor temperature controlled using a preheater considerably affects CH${}_{4}$ generation and transport. Although the usage of a high conductor temperature allows cable manufacturers to use high production speeds, the high conductor temperature increases the amount of CH${}_{4}$ generated from the cross-linking reaction because the β-scission reaction becomes more dominant than the hydrogen abstraction reaction. In addition, the high production speed significantly limits the amount of CH${}_{4}$ that can be removed from the cable. Simulations show that at similar production speeds, a higher conductor temperature results in less CH${}_{4}$ remaining in the cable. Approximately 2.7% less CH${}_{4}$ remains in the cable when the preheater is set at 160 °C compared with that when no preheater is used. Thus, a high cable temperature enhances both the CH${}_{4}$ generation and transport processes; however, its effect on CH${}_{4}$ transport is more significant. Another parameter considered in this study is the curing cube temperature distribution. In this study, we considered three temperature distribution strategies: constant temperature, linear incremental, and decremental temperature. The curing tube temperature distribution also affects the remaining CH${}_{4}$ concentration in the cable because CH${}_{4}$ generation and transport are considerably dependent on the temperature. The amount of CH${}_{4}$ remaining in the cable when the temperature was linearly increased from 300 °C to 400 °C was 1.6% and 3.7% lower than those observed when the temperature was maintained constant at 350 °C or when the temperature was linearly decreased from 400 °C to 300 °C, respectively. Further, we consider the effect of increasing the transition zone length and applying thermal insulation on the transition zone wall. Simulations indicate that increasing the transition zone length, which provides additional residence time for CH${}_{4}$ removal, and applying thermal insulation to the transition zone wall, which slows the decrease in cable temperature, enhance CH${}_{4}$ removal from the cable. The case studies indicate that the combination of these two factors increases CH${}_{4}$ removal from the cable by up to 9.7%. Finally, we consider the manner in which an additional heating zone, commonly referred to as the online relaxation section and located in the middle of the cooling section, influences CH${}_{4}$ removal from the cable. Results indicate that the online relaxation section minorly affects the remaining concentration of CH${}_{4}$ in the cable because the high temperature near the cable surface in this section can facilitate CH${}_{4}$ removal only within the area while more CH${}_{4}$ is still stored within the inner side of the cable. However, this effect on reducing CH${}_{4}$ concentration in the cable can increase if lower production speeds are applied, and thus, the residence time is extended.

## Figures and Tables

**Table 1:** CV line dimensions [5,30].

Section	Curing Tube	Transition Zone	Water Cooling	Air Cooling
Length (m)	36	4	82	115

**Table 2:** CV line operating condition.

Parameter	Cable Inlet Temp.	Water Flow Rate	Water Inlet Temp.
Operating condition	130 °C	3 m/h	25 °C

**Table 3:** Reaction kinetic parameters [31,33,34].

Parameter	Cross-Linking	Hydrogen Abstraction	β-Scission
		(Route *a*)	(Route *b*)
*A*	9.24 × $10^{15}$ s${}^{-1}$	1.35 × $10^{7}$ L·mol${}^{-1}\cdot$s${}^{-1}$	2.29 × $10^{12}$ s${}^{-1}$
$E_{a}$	152.67 kJ·mol${}^{-1}$	1.8 kJ·mol${}^{-1}$	35.9 kJ·mol${}^{-1}$

**Table 4:** Thermal and mechanical properties of the cable [4,5,28,30].

Property	Copper	XLPE	Semiconductor (SC)
Density, ρ (kg/m${}^{3}$)	8960	922	1050
Specific heat capacity, $C_{p}$ (J·kg${}^{-1}\cdot$K${}^{-1}$)	401	2700	1950
Thermal conductivity, *k* (W·m${}^{-1}\cdot$K${}^{-1}$)	385	0.335	0.53

**Table 5:** Case codes and parameters of conductor temperature studies.

Case Code	Conductor Temperature (°C)	Production Speed (m/min)
1a	160	2.8
1b	25	1.5
1c	160	1.5

**Table 6:** Remaining concentration of CH${}_{4}$ (mol/m) in each cable component at the cable take-up point for the study of the conductor temperature.

Case Code	Conductor	XLPE + SC	Entire Cable	Total CH${}_{4}$	%CH${}_{4}$
1a	0.0050	0.0833	0.0883	0.1125	19.2
1b	0.0058	0.0683	0.0741	0.1095	0.0
1c	0.0075	0.0646	0.0721	0.1126	−2.7

**Table 7:** Case codes and parameters of curing tube temperature distribution studies.

Case Code	Curing-Tube Temperature Distribution
2a	Linear increase from 300 °C to 400 °C
2b	Constant temperature of 350 °C
2c	Linear decrease from 400 °C to 300 °C

**Table 8:** Remaining concentration of CH${}_{4}$ (mol/m) in each cable component at the cable take-up point for the study of the curing tube temperature distribution.

Case Code	Conductor	XLPE + SC	Entire Cable	Total CH${}_{4}$	%CH${}_{4}$
2a	0.0056	0.0735	0.0791	0.1081	0.0
2b	0.0059	0.0745	0.0804	0.1118	1.6
2c	0.0063	0.0757	0.0820	0.1169	3.7

**Table 9:** Case codes and parameters of the transition zone study.

Case Code	Transition Zone Length (m)	Thermal Insulation at Transition Zone Wall
3a	4	No
3b	4	Yes
3c	8	No
3d	8	Yes
3e	20	No
3f	20	Yes

**Table 10:** Remaining concentration of CH${}_{4}$ (mol/m) in each cable component at the cable take-up point for the study of the transition zone.

Case Code	Conductor	XLPE + SC	Entire Cable	Total CH${}_{4}$	%CH${}_{4}$
3a	0.0056	0.0735	0.0791	0.1081	0.0
3b	0.0056	0.0731	0.0787		−0.5
3c	0.0059	0.0716	0.0775		−2.0
3d	0.0060	0.0706	0.0766		−3.2
3e	0.0066	0.0672	0.0738		−6.7
3f	0.0069	0.0645	0.0714		−9.7

**Table 11:** CV line parameters considered for the online relaxation study.

CV Line Code	Curing Tube (m)	Transition Zone (m)	1st Water -Cooling (m)	Online Relaxation (m)	2nd Water -Cooling (m)	Air -Cooling (m)	Total (m)
CVa	36	4	82	10	70	115	317
CVb			152	NA	NA		307

**Table 12:** Case codes and parameters of the online relaxation study.

Case Code	CV Line Code	Average Curing Tube Temperature (°C)	Production Speed (m/min)	Online Relaxation Temperature (°C)
4a	CVa	350	2.0	345
4b	CVb			NA
4c	CVa	250	1.1	320
4d	CVb			NA

**Table 13:** Remaining concentration of CH${}_{4}$ (mol/m) in each cable component at the cable take-up point for the study of online relaxation.

Case Code	Conductor	XLPE+SC	Entire Cable	Total CH${}_{4}$	%CH${}_{4}$
4a	0.0059	0.0734	0.0793	0.1118	−1.4
4b	0.0059	0.0745	0.0804		0.0
4c	0.0053	0.0632	0.0685	0.0960	−2.2
4d	0.0052	0.0648	0.0700		0.0

## Abbreviations

The following abbreviations are used in this manuscript:

AP	Acetophenone
CA	Cumyl alcohol
CH${}_{4}$	Methane
CV	Continuous vulcanization
DCP	Dicumyl peroxide
EHV	Extra high voltage
HV	High voltage
PE	Polyethylene
SC	Semiconductor
XLPE	Cross-linked polyethylene
%CH${}_{4}$	Fractional comparison between the cases regarding the remaining CH${}_{4}$ in the entire cable
